# Benefits of lung-protective ventilation: looking beyond the ICU

**DOI:** 10.1186/s13054-014-0530-0

**Published:** 2014-09-25

**Authors:** Namita Gupta, Sachin Yende

**Affiliations:** CRISMA Center, Department of Critical Care Medicine, University of Pittsburgh, 606D Scaife Hall, 3550 Terrace Street, Pittsburgh, PA 15261 USA; The Clinical Research, Investigation, and Systems Modeling of Acute Illness (CRISMA) Center, University of Pittsburgh, Pittsburgh, PA 15210 USA

## Expanded abstract

### Citations

Futier E, Constantin JM, Paugam-Burtz C, Pascal J, Eurin M, Neuschwander A, Marret E, Beaussier M, Gutton C, Lefrant JY, Allaouchiche B, Verzilli D, Leone M, De Jong A, Bazin JE, Pereira B, Jaber S; IMPROVE Study Group: **A trial of intraoperative low-tidal-volume ventilation in abdominal surgery.***N Engl J Med* 2013, **369:**428–437.

### Background

Lung-protective ventilation with the use of low tidal volumes and positive end-expiratory pressure is considered best practice in the care of many critically ill patients. However, its role in anesthetized patients undergoing major surgery is not known.

### Methods

*Objective:* The aim of the study was to determine whether lung-protective ventilation improves outcomes in anesthetized patients undergoing major abdominal surgery.

*Design:* The Intraoperative Protective Ventilation (IMPROVE) trial is a multicenter, open-label double-blind, parallel-group randomized control trial in seven French university hospitals.

*Setting:* The IMPROVE study enrolled 400 adults at intermediate to high risk of pulmonary complications undergoing major abdominal surgeries between 31 January 2011 and 10 August 2012.

*Intervention:* Patients were randomly assigned to receive volume-controlled ventilation in one of two strategies: nonprotective ventilation with a tidal volume of 10 to 12 ml/kg predicted body weight with no positive end-expiratory pressure and no scheduled recruitment maneuver, or lung-protective ventilation with a tidal volume of 6 to 8 ml/kg predicted body weight, a positive end-expiratory pressure of 6 to 8 cmH_2_O, and recruitment maneuvers every 30 minutes after intubation. Recruitment maneuvers were also standardized and applied as continuous positive airway pressure of 30 cmH_2_O for 30 seconds.

*Outcomes:* The primary outcome was a composite of major pulmonary and extrapulmonary complications within the first 7 days after surgery. Major pulmonary complications were defined as pneumonia or the need for invasive or noninvasive ventilation for acute respiratory failure. Major extrapulmonary complications were defined as sepsis, severe sepsis, septic shock, and death. Secondary outcomes included components of primary outcome, surgical complications, and healthcare utilization endpoints such as the duration of stay in the ICU and hospital at the end of a 30-day follow-up period.

### Results

The two intervention groups had similar characteristics at baseline. In the intention-to-treat analysis, the primary outcome occurred in 21 of 200 patients (10.5%) assigned to lung-protective ventilation, as compared with 55 of 200 patients (27.5%) assigned to nonprotective ventilation (relative risk, 0.40; 95% confidence interval, 0.24 to 0.68; P = 0.001). Over the 7-day postoperative period, 10 patients (5.0%) assigned to lung-protective ventilation required noninvasive ventilation or intubation for acute respiratory failure, as compared with 34 patients (17.0%) assigned to nonprotective ventilation (relative risk, 0.29; 95% confidence interval, 0.14 to 0.61; P = 0.001). The length of the hospital stay was shorter among patients receiving lung-protective ventilation than among those receiving nonprotective ventilation (mean difference, −2.45 days; 95% confidence interval, 4.17 to −0.72; P = 0.006).

### Conclusions

As compared with a practice of nonprotective mechanical ventilation, the use of a lung-protective ventilation strategy in intermediate-risk and high-risk patients undergoing major abdominal surgery was associated with improved clinical outcomes and reduced healthcare utilization.

## Commentary

Postoperative pulmonary complications after surgery are common (2 to 19%), serious, and expensive [1-3]. The effect of intraoperative mechanical ventilation on postoperative pulmonary complications in patients undergoing general anesthesia is poorly understood. Most patients are ventilated for a brief duration in the operating room. Thus, little attention has been focused on ventilation strategies in the operating room. Lung-protective ventilation using low tidal volumes (TV) and low plateau pressure has shown clinical benefit in critically ill patients, both with and without acute respiratory distress syndrome [4,5], but its role in the operating room is unclear.

To date, several trials testing lung-protective ventilation during surgery have shown conflicting results. Most of these trials were small and used surrogate endpoints, such as biomarkers of pulmonary and systemic inflammation [6-11]. A recent meta-analysis by Hemmes and colleagues examined the role of perioperative use of lower TV, higher levels of positive end-expiratory pressure (PEEP), and recruitment maneuvers (RM) in noncardiac surgical patients since 1966 [12]. The included trials used varying levels of PEEP, and RM were used inconsistently. The authors concluded that intraoperative use of low TV may reduce postoperative lung injury, pneumonia, and atelectasis. Whether higher levels of PEEP with or without RM added to the beneficial effects remained uncertain, however, underscoring the need for large randomized controlled trials to rigorously test the effect of lung-protective ventilation.

The Intraoperative Protective Ventilation (IMPROVE) trial addressed this need by comparing a lung-protective ventilation strategy, combining low TV, PEEP, and RM, with conventional ventilation. This study was a prospective, randomized, open-label, double-blind trial. The authors chose to focus on patients undergoing elective abdominal surgery with a longer duration because these patients are at high risk for postoperative pulmonary complications. The intervention arm of the study used TV of 6 to 8 ml/kg predicted body weight, PEEP of 6 to 8 cmH_2_O, and RM repeated every 30 minutes after tracheal intubation. The control group used TV of 10 to 12 ml/kg and no PEEP, and RM were performed at the physician’s discretion. The primary outcome assessed at 7 days was a composite of major pulmonary and nonpulmonary complications, occurring in 10.5% of patients assigned to lung-protective ventilation compared with 27.5% assigned to the control arm (*P* = 0.001). These differences were largely driven by differences in the risk of pneumonia (1.5% versus 8%), need for non-invasive ventilation (4.5% versus 14.5%), and sepsis (6.5% versus 14.5%). These differences persisted at 30 days, as evidenced by higher risks for pneumonia, need for invasive and non-invasive mechanical ventilation, and sepsis. Additionally, patients receiving lung-protective ventilation had fewer surgical complications, including anastamotic leakage (12% versus 22%) and shorter hospital stay (mean difference −2.45 days; *P* = 0.006). There were no differences in intervention-related adverse events such as intraoperative hypotension, in pneumothorax, or in 30-day mortality. The authors thus concluded that the use of a lung-protective ventilation strategy in intermediate-risk and high-risk patients undergoing major abdominal surgery was associated with improved clinical outcomes and reduced healthcare utilization.

The rationale behind this study rests on unequivocal evidence that mechanical ventilation has the potential to precipitate ventilator-induced lung injury mainly via volutrauma, atelectrauma, barotrauma, and biotrauma in both healthy and injured lungs [13]. Although perioperative use of mechanical ventilation is for shorter duration followed by a rapid wean, the potential for ventilator-induced lung injury exists. Induction of general anesthesia, especially use of muscle relaxants, promotes atelectasis in 90% of patients [14]. These changes occur within minutes after induction, and often persist for several days after surgery. Furthermore, experimental work and some clinical studies suggest that atelectasis of the lung increases bacterial growth and may lead to pneumonia [15]. To reduce atelectasis, anesthesiologists have historically applied large TV. This strategy, however, can overdistend nondependent lung tissue and cause volutrauma. Zero PEEP or PEEP less than 5 cmH_2_O are used in about 80% of intraoperatively ventilated patients and RM are used in less than 10% of patients [16]. Repeated opening and closing of alveoli in the presence of low PEEP and the absence of RM can potentially lead to shear stress causing atelectrauma. Several findings of this trial suggest that atelectrauma, volutrauma, and biotrauma may be minimized by lung-protective ventilation. For example, the respiratory system compliance at the end of surgery was higher in the intervention arm (55.2 ± 26.7 versus 45.1 ± 12.9 ml/cmH_2_O). Invasive or non-invasive ventilation postoperatively is often required for atelectasis and was used less frequently in the intervention group.

The main limitation of the study is the use of TV greater than 10 ml/kg with zero PEEP in the control arm, which may not represent common practices in the operating room around the world. Almost a decade after the Acute Respiratory Distress Syndrome Network results, several observational studies have shown a decline in average TV [17]. Whether the positive outcome in the study is as a result of their intervention or is secondary to a control arm that does not represent the best known practices remains uncertain. The study also excluded obese patients with a body mass index greater than 35 kg/m^2^ who in observational studies are at a risk of receiving higher TV and are more prone to developing atelectasis, and in whom a protective strategy of ventilation may be more useful.

## Recommendation

The findings of the IMPROVE study emphasize the importance of intraoperative lung-protective ventilation on perioperative morbidity, and even transient exposure can show significant improvement of outcomes. The results also further add to a strong body of evidence that ventilating at high TV has clinically meaningful deleterious effects and that conscience efforts should be taken to abandon the one-tidal-volume fits-all approach.

## Abbreviations

IMPROVE: Intraoperative Protective ventilation
PEEP: Positive end-expiratory pressure
RM: Recruitment maneuvers
TV: Tidal volumes

## References

[1] Johnson RG, Arozullah AM, Neumayer L, Henderson WG, Hosokawa P, Khuri SF (2007). Multivariable predictors of postoperative respiratory failure after general and vascular surgery: results from the patient safety in surgery study. J Am Coll Surg.

[2] Finks JF, Osborne NH, Birkmeyer JD (2011). Trends in hospital volume and operative mortality for high-risk surgery. N Engl J Med.

[3] Dimick JB, Chen SL, Taheri PA, Henderson WG, Khuri SF, Campbell DA (2004). Hospital costs associated with surgical complications: a report from the private-sector National Surgical Quality Improvement Program. J Am Coll Surg.

[4] Acute Respiratory Distress Syndrome Network (2000). Ventilation with lower tidal volumes as compared with traditional tidal volumes for acute lung injury and the acute respiratory distress syndrome. N Engl J Med.

[5] Serpa NA, Cardoso SO, Manetta JA, Pereira VG, Espósito DC, Pasqualucci Mde O, Damesceno MC, Schultz MJ (2012). Association between use of lung-protective ventilation with lower tidal volumes and clinical outcomes among patients without acute respiratory distress syndrome: a meta-analysis. JAMA.

[6] Wrigge H, Zinserling J, Stuber F, von Spiegel T, Hering R, Wetegrove S, Hoeft A, Putensen C (2000). Effects of mechanical ventilation on release of cytokines into systemic circulation in patients with normal pulmonary function. Anesthesiology.

[7] Wrigge H, Uhlig U, Zinserling J, Behrends-Callsen E, Ottersbach G, Fischer M, Uhlig S, Putensen C (2004). The effects of different ventilatory settings on pulmonary and systemic inflammatory responses during major surgery. Anesth Analg.

[8] Wolthuis EK, Choi G, Dessing MC, Bresser P, Lutter R, Dzoljic M, van der Poll T, Vroom MB, Hollmann M, Schultz MJ (2008). Mechanical ventilation with lower tidal volumes and positive end-expiratory pressure prevents pulmonary inflammation in patients without preexisting lung injury. Anesthesiology.

[9] Treschan TA, Kaisers W, Schaefer MS, Bastin B, Schmalz U, Wania V, Eisenberger CF, Saleh A, Weiss M, Schmitz A, Kienbaum P, Sessler DI, Pannen B, Beiderlinden M (2012). Ventilation with low tidal volumes during upper abdominal surgery does not improve postoperative lung function. Br J Anaesth.

[10] Weingarten TN, Whalen FX, Warner DO, Gajic O, Schears GJ, Snyder MR, Schroeder DR, Sprung J (2010). Comparison of two ventilatory strategies in elderly patients undergoing major abdominal surgery. Br J Anaesth.

[11] Severgnini P, Selmo G, Lanza C, Chiesa A, Friegerio A, Bacuzzi A, Dionigi G, Novario R, Geroretti C, de Abreu MG, Schultz MJ, Jaber S, Futier E, Chiaranda M, Pelosi P (2013). Protective mechanical ventilation during general anesthesia for open abdominal surgery improves postoperative pulmonary function. Anesthesiology.

[12] Hemmes SN, Serpa NA, Schultz MJ (2013). Intra operative ventilatory strategies to prevent postoperative pulmonary complications: a meta-analysis. Curr Opin Anaesthesiol.

[13] Slutsky AS, Ranieri VM (2013). Ventilator-induced lung injury. N Engl J Med.

[14] Tusman G, Bohm SH, Warner DO, Sprung J (2012). Atelectasis and perioperative pulmonary complications in high-risk patients. Curr Opin Anaesthesiol.

[15] van Kaam AHLC, Lutter R, Lachmann RA, Haitsma JJ, Herting E, Snoek M, De Jaegere A, Kok JH, Lachmann B (2005). Effect of ventilation strategy and surfactant on inflammation in experimental pneumonia. Eur Respir J.

[16] Jaber S, Coisel Y, Chanques G, Futier E, Constantin JM, Michelet P, Beaussier M, Lefrant JY, Allaouchiche B, Capdevila X, Marret E (2012). A multicentre observational study of intra-operative ventilatory management during general anaesthesia: tidal volumes and relation to body weight. Anaesthesia.

[17] Esteban A, Frutos-Vivar F, Muriel A, Ferguson ND, Peñyelas O, Abraira V, Raymondos K, Rios F, Nin N, Apeztequía C, Violi DA, Thille AW, Brochard L, González M, Villagomez AJ, Hurtado J, Davies AR, Du B, Maggiore SM, Pelosi P, Soto L, Tomicic C, D’Empaire G, Matamis D, Abroug F, Moreno RP, Soares MA, Arabi Y, Sandi F, Jibaja M (2013). Evolution of mortality over time in patients receiving mechanical ventilation. Am J Respir Crit Care Med.

